# From Dyes to Drugs? Selective Leishmanicidal Efficacy of Repositioned Methylene Blue and Its Derivatives in In Vitro Evaluation

**DOI:** 10.3390/biology14121709

**Published:** 2025-11-30

**Authors:** Deyvison Rhuan Vasco-dos-Santos, Juliana Almeida-Silva, Ludmila Ferreira de Almeida Fiuza, Natalia Vacani-Martins, Zênis Novais da Rocha, Maria de Nazaré Correia Soeiro, Andrea Henriques-Pons, Eduardo Caio Torres-Santos, Marcos André Vannier-Santos

**Affiliations:** 1 Laboratório de Inovação em Terapias, Ensino e Bioprodutos, Instituto Oswaldo Cruz (LITEB—IOC), Fundação Oswaldo Cruz (Fiocruz), Rio de Janeiro 21040-900, RJ, Brazil; jualmeida@yahoo.com (J.A.-S.); natalia.vacani1989@gmail.com (N.V.-M.); andreah@ioc.fiocruz.br (A.H.-P.); 2 Laboratório de Biologia Celular, Instituto Oswaldo Cruz (LBC—IOC), Fundação Oswaldo Cruz (Fiocruz), Rio de Janeiro 21040-900, RJ, Brazil; ludmilafiuza93@gmail.com (L.F.d.A.F.); soeiro@ioc.fiocruz.br (M.d.N.C.S.); 3 Instituto de Química, Universidade Federal da Bahia (UFBA), *Campus* Universitário de Ondina, Salvador 40170-290, BA, Brazil; zenis@ufba.br; 4 Laboratório de Bioquímica de Tripanossomatídeos, Instituto Oswaldo Cruz (LBqT—IOC), Fundação Oswaldo Cruz (Fiocruz), Rio de Janeiro 21040-900, RJ, Brazil; ects@ioc.fiocruz.br

**Keywords:** cutaneous leishmaniasis, *Leishmania amazonensis*, chemotherapy, methylene blue, ruthenium complexes, selectivity, cell death

## Abstract

Chemotherapy for cutaneous leishmaniasis is hindered by high toxicity, adverse effects, and increasing drug resistance. Thus, safer and more selective therapies are urgently required. Here, we evaluated the antiparasitic efficacy of methylene blue (MB) and new MB, as well as novel ruthenium complex derivatives (NMB-B and NMB-P) against promastigote and amastigote forms of *Leishmania amazonensis*. Their cytotoxicity and selectivity on L929, HepG2, VERO, J774.G8 cells, and murine peritoneal macrophages were measured. Mechanisms of action were explored via flow cytometry, assessing morphological changes, mitochondrial depolarization, ROS production, and cell death. The compounds inhibited parasite proliferation in a dose and time-dependent manner, achieving submicromolar efficacy against amastigotes (NMB-P = 0.46 μM). No cytotoxicity was observed on L929, J774.G8, and VERO cells (except NMB), while HepG2 and murine peritoneal macrophages showed low to moderate toxicity. Selective indexes reached 84 for promastigotes and over 500 for amastigotes. The compounds induced mitochondrial depolarization by up to 61% and a five-fold increase in ROS levels, leading to structural damage and parasite death via late apoptosis/necrosis-like mechanisms. These findings indicate that the compounds act selectively and trigger the release of oxidative species, exerting leishmanicidal activity and warranting further investigation.

## Simple Summary

Cutaneous leishmaniasis is an insect-borne disease caused by microscopic parasites called *Leishmania*, which enter the body cells and can cause sores, including on the face. Used treatments are mainly old, painful, and can harm the liver, kidneys, and heart. Therefore, it is important to discover novel effective, safe, and orally administered medications. In this study, we tested methylene blue, the first fully synthetic drug used in medicine. This vintage compound was previously used for blood disorders, including malaria, and is presently being evaluated for other conditions. Here, we approached the antiparasitic activity of methylene blue and its derivatives upon *Leishmania* and tested whether they affected laboratory-grown cells. We found that the substances effectively diminished parasite survival without exerting toxic effects on skin cells and with only low to moderate toxicity to liver and kidney cells. It is noteworthy that methylene blue can be protective for heart and nerve tissues. These data suggest that the tested substances are promising for the development of treatments for cutaneous leishmaniasis.

## 1. Introduction

Leishmaniasis encompasses a group of tropical neglected diseases, also known as the diseases of poverty, being intrinsically linked to malnutrition and socioeconomic vulnerabilities, with an estimated one billion individuals living at a risk of infection [1,2,3,4]. These diseases are caused by the protozoa of the genus *Leishmania*, transmitted by phlebotomine sandflies [5,6]. Recent studies have also demonstrated the possibility of oral and intragastric transmission in experimental hamster infection [7]. Despite the spectral clinical manifestations in humans, there are three main forms of leishmaniasis: visceral, cutaneous, and mucocutaneous [8,9].

In the Americas, cutaneous leishmaniasis (CL) is the most prevalent form, caused by various species including, *Leishmania amazonensis*. The complications range from ulcers to immunopathogenic syndromes, potentially escalating to visceral leishmaniasis depending on the parasite virulence and the patient’s immunocompetence [10,11,12]. CL can also result in permanent and disfiguring lesions, leading to social stigmatization, psychological distress, and decreased work capacity and quality of life [13,14,15].

The therapeutic arsenal for leishmaniasis remains limited, with pentavalent antimonials such as meglumine antimoniate (Glucantime^®^) and sodium stibogluconate (Pentostam^®^) being the primary choice for nearly seven decades [16,17,18]. These medications are associated with severe adverse effects, including myalgia, arthralgia, pancreatitis, and gastrointestinal and respiratory disorders [19,20]. They are also cardio [21,22,23], hepato- [24], geno- [25], neuro- [26,27], and nephrotoxic [28,29], making them contraindicated for groups such as pregnant [30] and renal patients [31].

Additionally, the administration of antimonials via parenteral routes over extended periods is associated with significant pain, hospitalization requirements, reduced patient adherence, and treatment continuity, leading to therapeutic failures [32,33,34] and resistance phenotype selection [35]. Hence, there is a pressing need for the development of new therapies that are not only accessible and well tolerated but also can be administered orally or topically and require brief treatment courses [36,37].

Drug repositioning emerges as an advantageous strategy among the available alternatives. This approach utilizes existing drugs with well-established pharmacokinetic and safety profiles, effectively reducing development time, costs, and associated risks. As a result, it potentially shortens the timeline from drug development to patient access, ensuring the faster availability of treatments, particularly for those relying on public healthcare systems [38,39]. Examples of repurposed drugs for testing in *Leishmania* species include methylene blue (MB), the first synthetic substance used as a medication [40]. This vintage drug is still approached for distinct disorders and may be repositioned for conditions such as Alzheimer’s disease [41], acute liver failure [42], and COVID-19 [43].

Considering the aforementioned cardio- and neurotoxicity of antileishmanial drugs, which may be cumulative [44], it is important to point out that MB can display neuro- and cardioprotective effects [45]. The neuroprotection is relevant not only for the therapy that often requires multiple treatment courses, but also because *Leishmania* spp. infection is repeatedly associated with neurological manifestations in humans [46,47] and other animals [48], including perineural inflammation [49]. In this regard, it is interesting that MB is anti-inflammatory [50], able to inhibit neuroinflammation [51].

MB presents low cost, high hydrosolubility [52], and has been applied as an antimalarial for over a century [53]. Its antileishmanial action is associated with its potential as a photosensitizer in photodynamic therapy (PDT) [54,55,56], inducing mitochondrial damage [57], with effects observed in CL caused by *L. amazonensis* in experimental murine [58] and hamster [59] infections as well as in human cases [60,61]. However, studies elucidating the antileishmanial activity of MB without photostimulation are incipient.

Thus, we evaluated the in vitro activity of MB, new methylene blue (NMB), and its ruthenium complexes (new methylene blue B—NMB-B and new methylene blue P—NMB-P) against promastigote and ex vivo amastigote forms of *L. amazonensis*, without light exposure. Ruthenium complexes have been described as catalysts of potent and synergistic leishmanicidal activities [62,63,64,65], encouraging the investigation of NMB-B and NMB-P complexes.

In this study, we also investigated the cytotoxicity and selectivity of the compounds in different mammalian cells. Evidence suggests that the screening of drug candidates considering pharmacokinetics and pharmacodynamics is more effective when performed in multiple host cell types [66]. We have previously noticed that another ruthenium complex was fungicidal and triggered lipid peroxidation, damaging the stable cell walls of fluconazole-resistant *Candida tropicalis*, even with no photostimulation [67]. Therefore, we hypothesize that the compounds tested here can display promising leishmanicidal activity, even without photodynamic dynamization.

## 2. Material and Methods

### 2.1. Compounds and Reagents

**Figure 1:**
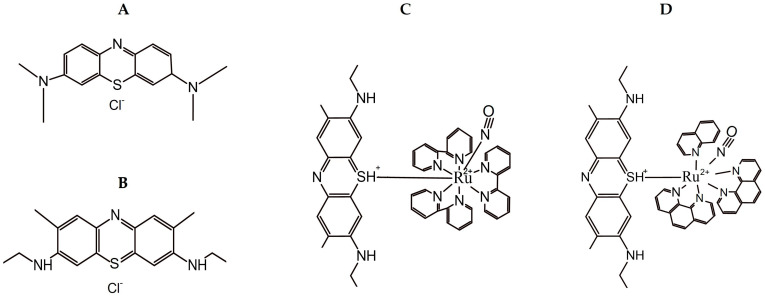
Molecular structure of methylene blue (MB—(**A**)), new methylene blue (NMB—(**B**)), new methylene blue B (NMB-B—(**C**)), and new methylene blue P (NMB-P—(**D**)). The structures were constructed using ChemSketch (Freeware) 2023.2.4.

MB, NMB (Figure 1A,B), and miltefosine (MT) were acquired from Sigma-Aldrich (St. Louis, MO, USA), as well as ruthenium trichloride (RuCl_3_·nH_2_O), 2,2′-bipyridine (bpy), and 1,10-phenanthroline (phen), which were utilized in the synthesis of NMB-B and NMB-P complexes (Figure 1C,D). These complexes were synthesized by the Instituto de Química da Universidade Federal da Bahia (UFBA—Salvador, BA, Brazil). MT was dissolved in sterile water and the other compounds in phosphate-buffered saline (PBS; Gibco—Grand Island, NY, USA). All substances were stored at −20 °C until use.

Schneider’s Insect Medium and Dulbecco’s Modified Eagle Medium (DMEM), penicillin/streptomycin (Pen/Str), L-glutamine, carbonyl cyanide 4-(trifluoromethoxy)phenylhydrazone (FCCP), Antimycin A (AA), RPMI 1640 without phenol, and brewer’s thioglycolate were obtained from Sigma-Aldrich (St. Louis, MO, USA). Fetal bovine serum (FBS) from Cultilab (Campinas, SP, Brazil) and Alamar Blue (AB) from Invitrogen (Eugene, OR, USA). FITC Annexin V Apoptosis detection Kit I and was purchased from BD Pharmingen (Franklin Lakes, NJ, USA); 7-Amino-Actinomycin D (7-AAD) from BioLegend (San Diego, CA, USA); tetramethylrhodamine ethyl (TMRE) from Molecular Probes (Carlsbad, CA, USA); and 2′,7′-dichlorodihydrofluorescein diacetate (H_2_DCFDA) from PROC9 (Canoas, RS, Brazil).

### 2.2. Synthesis of Ruthenium Complexes

The target complexes were synthesized starting with RuCl_3_·nH_2_O as the primary material. The complexes cis-[RuCl_2_(X-Y)_2_] (where X-Y represents polypyridyl ligands such as phen and bpy) and cis-[Ru(NO_2_)_2_(X-Y)_2_](PF_6_)_2_ were intermediates in the preparation of the final complexes, cis-[Ru(NO)(NMB)(X-Y)_2_](PF_6_)_4_. To prepare these complexes, 0.05 g (0.094 mmol) of [RuCl_2_(X-Y)_2_] was dissolved in a 5 mL/5 mL ethanol/water mixture under an argon atmosphere and heated at 55 °C for 15 min. Subsequently, 0.035 g (~0.094 mmol) of NMB and 0.033 g (0.0187 mmol) of potassium nitrate were added. For all systems, the mixture was maintained under stirring and argon atmosphere at 55 °C for 2 h, followed by the addition of 100 mg of NH_4_PF_6_ (0.61 mmol) and 1 mL of HPF_6_ 0.1 mol L^−1^ solutions. The resulting green precipitation was collected through filtration.

### 2.3. Ethical and Animal Statements

Access to genetic resources was granted by the National System for Genetic Heritage and Associated Traditional Knowledge (Sistema Nacional de Gestão do Patrimônio Genético e do Conhecimento Tradicional Associado—SisGen) under registration AD1C828. All animal study protocols were approved by the Comitê de Ética para o Uso de Animais (CEUA—IOC/Fiocruz) in Rio de Janeiro (lic. L038-2017 A4) and comply with the guidelines of the National Council for the Control of Animal Experimentation (Conselho Nacional de Controle de Experimentação Animal—CONCEA).

### 2.4. Culture of Parasitic Cells

Promastigote forms of *Leishmania amazonensis* (IFLA/BR/1967/PH8) were obtained from the Instituto Oswaldo Cruz *Leishmania* collection (CLIOC/Fiocruz). These cultures were maintained in Schneider’s Insect Medium, supplemented with 20% FBS and 1 μL/mL of Pen/Str at 26 °C. In vitro passages were conducted weekly, with promastigotes harvested during the late logarithmic phase for experiments on the 5th day of growth [68] (Figure S1A).

Ex vivo amastigote forms of *L. amazonensis* (MHOM/BR/77/LTB0016) were harvested and purified from lesions of male BALB/c mice. For this, a suspension containing 10^6^ amastigotes was inoculated subcutaneously into the intraplantar region of the hind paw. After 30 days, the animals were euthanized, and their paws were excised and rinsed in 70% ethanol. The epidermis and necrotic tissues were mechanically dissociated. Subsequently, the paws were placed in Petri dishes containing RPMI 1640 medium supplemented with 5% FBS and 2% Pen/Str. To liberate the amastigotes, the tissues were scraped, and the suspension was transferred to conical tubes, vigorously homogenized using a 20 mL syringe, and centrifuged at 101× *g* for 15 min. The supernatant was transferred to another tube, homogenized with a syringe, and centrifuged at 764× *g* for another 15 min. The supernatant was discarded, and the pellet was resuspended in RPMI 1640 medium [69].

### 2.5. Culture of Mammalian Host Cells

HepG2 cells (human hepatocellular carcinoma), VERO cells (African green monkey kidney), and J774.G8 macrophages (murine reticulosarcoma) were maintained in DMEM medium, supplemented with 10% FBS, 1% Pen/Str at 37 °C and 5% CO_2_ [70,71]. For the cultivation of L929 fibroblasts (murine fibrosarcoma), RPMI 1640 medium without phenol red, supplemented with 2 mM L-glutamine, 10% FBS, and 1% Pen/Str, was used and maintained under the same conditions of temperature and CO_2_ as above [72].

Murine peritoneal macrophages—MPMs (primary cells)—were isolated from male Swiss Webster mice stimulated with 1 mL of 3% Brewer’s thioglycollate medium, administered intraperitoneally. After 96 h, the animals were euthanized, and the MPMs were collected by peritoneal lavage with approximately 10 mL of RPMI 1640 medium. The cell suspension was centrifuged at 382× *g* for 10 min, and the pellet was resuspended in medium supplemented with 5% FBS, 1% Pen/Str, and 2 mM L-glutamine. All cell cultures were maintained at 37 °C and 5% CO_2_ [73]. For the experiments, all cell types were cultured until confluency.

### 2.6. Leishmanicidal Activity Assay: Evaluation of Promastigotes and Ex Vivo Amastigotes

Promastigote and ex vivo amastigote forms of *L. amazonensis* (10^6^ parasites/mL) were seeded into 96-well flat-bottom microplates and treated with various concentrations of the compounds, serially diluted in Schneider/RPMI 1640 medium to a final volume of 100 µL/well. Promastigotes were kept at 26 °C, whereas amastigotes were kept at 32 °C. After 24 h, parasite viability was assessed using the AB colorimetric method, and the data were analyzed using a spectrophotometer as previously described [74]. The results were used to determine the 50% Maximal Inhibitory Concentration values (IC_50_). For promastigote forms, viability was also spectrophotometrically evaluated using AB through growth curves in the presence of the compounds at IC_50_ and different time points (24–168 h). Wells with medium and without parasites were used as blanks, and wells with medium and untreated parasites were used as negative controls. MT was employed as the reference drug.

### 2.7. Cytotoxicity Assays on Different Mammalian Cell Types and Selective Indexes

J774.G8, VERO (10^5^ cells/well), HepG2, L929 (0.5 × 10^5^ cells/well) cells, and MPMs (3 × 10^5^ cells/well) were centrifuged, resuspended in medium, plated in 96-well microplates, and incubated for 24 h under cultivation conditions. Subsequently, they were exposed to different concentrations of the compounds, serially diluted in medium to a final volume of 100 µL/well, and incubated for 24 h at 37 °C and 5% CO_2_. Cell viability was then assessed using the AB, and data were acquired by spectrophotometry [74]. The results were used to calculate the 50% Maximal Cytotoxicity Concentration (CC_50_). Untreated cells were used as negative controls, and cells exposed to the reference compound (MT) were used as positive controls. The Selectivity Index (SI) was calculated using the ratio SI = CC_50_ (mammalian cells)/IC_50_ (promastigote or amastigote forms of *L. amazonensis*).

### 2.8. Evaluation of Mechanisms of Action by Flow Cytometry

Initially, we treated promastigote forms of *L. amazonensis* (10^6^ parasites/mL) with 200 μM of MB, the highest concentration used in the inhibition assays, and with the IC_50_ of each compound. We observed that the samples did not emit fluorescence in the channels of interest (FITC, PE, and PerCP—Figure S2). Next, both promastigotes and ex vivo amastigotes were exposed to the IC_50_ of the compounds for 24 h. They were then evaluated for mitochondrial membrane potential (ΔΨm), reactive oxygen species (ROS) production (5 × 10^6^ parasites/mL), and detection of apoptosis/necrosis (10^5^ parasites/mL). Data acquisition was performed using a Cytoflex S flow cytometer (Beckman Coulter, Brea, CA, USA) at the Flow Cytometry Facility—Unity of Multiparametric Analysis of Instituto Oswaldo Cruz—and analyzed using the Cytexpert software (version 2.5). In total, 10,000 events were acquired in the regions corresponding to *L. amazonensis*, previously established.

#### 2.8.1. Determination of Mitochondrial Membrane Potential—ΔΨm

The parasites were incubated with 0.05 µM of TMRE at 28 °C for 30 min. Untreated parasites, labeled with 10 µM of FCCP and TMRE^+^, were used as positive controls. Untreated parasites labeled with TMRE were used as negative controls. TMRE labeling was evaluated by median fluorescence intensity (MFI) in the PE channel, from which the variation index of mitochondrial membrane potential (VI_ΔΨm_) was calculated using the equation: VI_ΔΨm_ = (MT − MC)/MC, where MT corresponds to the MFI of TMRE in treated parasites, and MC corresponds to the MFI of TMRE in control parasites. Negative results indicate mitochondrial membrane depolarization.

#### 2.8.2. Generation of Reactive Oxygen Species—ROS

For ROS production analysis, both parasite forms studied were labeled with 20 µM of H_2_DCFDA for 20 min at 28 °C in the dark. As a positive control, parasites were incubated with 10 µM of AA, while untreated parasites served as the negative control. The percentage of H_2_DCFDA labeling was measured in the FITC channel to calculate the variation index of ROS production (VI_ROS_) using the equation: VI_ROS_ = MT/MC, where MT corresponds to the H_2_DCFDA MFI of treated parasites, and MC corresponds to the H_2_DCFDA MFI of control parasites.

#### 2.8.3. Cell Death: Phosphatidylserine Exposure and Plasma Membrane Integrity

Cells were incubated with the FITC Annexin V Apoptosis Detection Kit I to detect apoptosis and necrosis. Additionally, for ex vivo amastigotes, labeling with pre-titrated 7-AAD probe was employed. All markers were used according to the manufacturers’ recommendations. Parasites heated to 60 °C for 15 min were used as positive controls, while untreated parasites were used as negative controls (Figure S3). The percentages of apoptotic and/or necrotic events were evaluated based on Annexin V (AV), propidium iodide (PI), and 7-AAD positivity in the FITC, PE, and PerCP channels, respectively.

#### 2.8.4. Image Flow Cytometry

Parasites treated as described above for the apoptosis/necrosis detection assay and labeled with Annexin FITC/PI were analyzed by image flow cytometry. Sample acquisition was performed using the Amnis^®^ ImageStream^®^X Mk II (Cytek^®^ Biosciences Inc., Fremont, CA, USA) and analyzed with IDEAS software (version 6.3). Samples were acquired with a typical setup, including a 40× objective and a numerical aperture 0.75. Detection was performed using side scatter, brightfield, and two fluorescence images. The flow cytometer was equipped with a 488 nm excitation laser, and band pass filters of 480–560 nm and 595–642 nm were used to evaluate Annexin FITC and PI labeling, respectively. The image size of the cytometer was 0.5 × 0.5 μm per pixel, with a field of view of 60 × 128 μm at an image generation rate of 500 cells per second. Single positive parasites (Annexin FITC^+^ or PI^+^) were used to create the compensation matrix and generate the images.

### 2.9. Statistical Analysis

All assays were performed at least twice in three independent replicates. Data obtained were plotted using Microsoft Excel (version 2406), and GraphPad Prism^®^ version 8 (GraphPad Software Inc., San Diego, CA, USA) was used to calculate IC_50_, CC_50_, and perform statistical analyses. One-way analysis of variance (ANOVA) followed by Dunnett’s post-test was used to compare groups. Differences were considered significant when *p* < 0.05 (*), *p* < 0.001 (**), and *p* < 0.0001 (***) as compared to the controls.

## 3. Results

### 3.1. Compounds Surpass the Efficacy of the Reference Drug in Inhibiting Different Forms of Leishmania amazonensis

**Figure 2:**
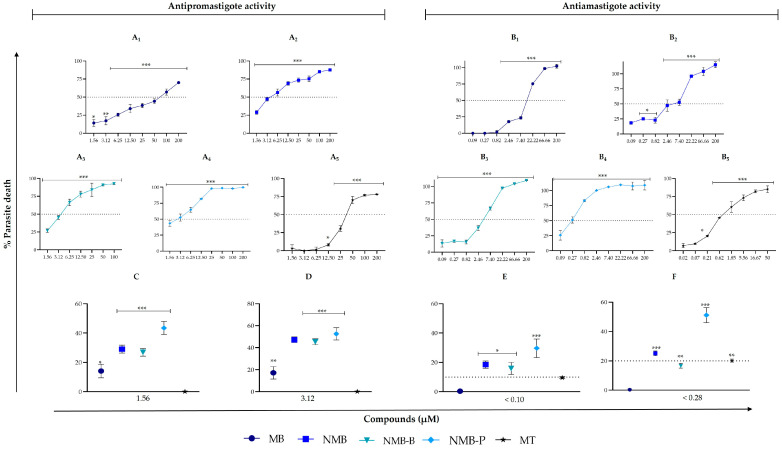
In vitro activity of methylene blue (MB), new methylene blue (NMB), new methylene blue B (NMB-B), new methylene blue P (NMB-P), and miltefosine (MT) on *Leishmania amazonensis* (10^6^ parasites/mL). Panel A: dose–response curves of MB (**A_1_**), NMB (**A_2_**), NMB-B (**A_3_**), NMB-P (**A_4_**), and MT (**A_5_**) against promastigote forms (*L. amazonensis* PH8 strain). Panel B: dose–response curves of MB (**B_1_**), NMB (**B_2_**), NMB-B (**B_3_**), NMB-P (**B_4_**), and MT (**B_5_**) against ex vivo amastigote forms (*L. amazonensis* LTB0016 strain). (**C**,**D**): Comparison between the compounds and MT at 1.56 μM (**C**) and 3.12 μM (**D**) on promastigote forms. (**E**,**F**) Comparison between the compounds and MT at <0.10 μM (**E**) and <0.28 μM (**F**) on ex vivo amastigote forms. Data represent mean ± SD. (*) *p* < 0.05; (**) *p* < 0.001; (***) *p* < 0.0001 when compared to negative control (untreated parasites) by one-way ANOVA and Dunnett’s post-test.

The inhibitory actions of MB, NMB, NMB-B, and NMB-P were evaluated on promastigote and ex vivo amastigote forms of *L. amazonensis*. All compounds reduced the viability of both parasitic forms in a concentration-dependent manner (Figure 2A_1_–B_5_) and significantly (*p* < 0.05) as compared to the negative control. Some compounds were more effective at lower concentrations than the reference drug, MT. In promastigotes, at 1.56 μM and 3.12 μM, all compounds caused greater antiparasitic effects than MT (Figure 2C,D). In ex vivo amastigotes, NMB, NMB-B, and NMB-P showed more inhibitory action at doses < 0.10 μM, while at concentrations below 0.28 μM, both NMB and NMB-P surpassed the efficacy of MT (Figure 2E,F). IC_50_ values were obtained from dose–response assays (Table 1).

NMB and ruthenium complexes stood out for promastigote forms with IC_50_ < 5.50 μM, especially NMB-P with an IC_50_ value of 2.84 ± 0.80 μM. These results were highly significant (*p* < 0.0001) compared to MT. Conversely, MB showed low leishmanicidal activity upon this developmental form (IC_50_ = 61.44 ± 4.41 μM). However, ex vivo amastigote forms were more susceptible to all tested compounds, with IC_50_ values reduced by 2- to 6-fold compared to promastigotes. NMB-P achieved submicromolar IC_50_ values (0.46 ± 0.34 μM), and other treatments demonstrated promising results.

**Figure 3:**
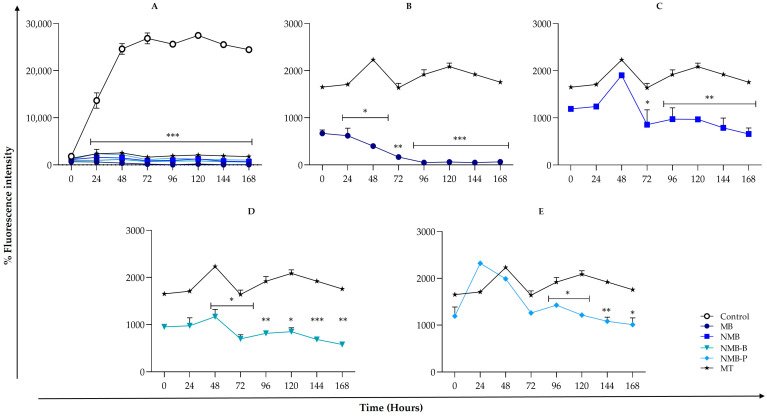
In vitro proliferation of *Leishmania amazonensis* promastigote forms (10^6^ parasites/mL, PH8 strain) exposed to IC_50_ of methylene blue (MB—61.44 µM), new methylene blue (NMB—5.42 µM), new methylene blue B (NMB-B—5.48 µM), new methylene blue P (NMB-P—2.84 µM), and miltefosine (MT—23.20 µM) over 168 h of incubation. (**A**) Fluorescence intensity comparison between untreated and treated parasites. (**B**–**E**) Fluorescence intensity over time for parasites treated with MB (**B**), NMB (**C**), NMB-B (**D**), and NMB-P (**E**), compared to MT. Data represent mean ± SD. (*) *p* < 0.05; (**) *p* < 0.001; (***) *p* < 0.0001 when compared to untreated parasites (**A**) and the standard drug MT (**B**–**E**) by one-way ANOVA and Dunnett’s post-test. IC_50_ = 50% Maximal Inhibitory Concentration.

Based on IC_50_ determinations (Table 1), we evaluated the survival and proliferation of promastigote forms incubated for 24 to 168 h (Figure 3). The growth curves showed highly significant (*p* < 0.0001) and time-dependent inhibition compared to the control at all periods analyzed (Figure 3A). Miltefosine showed more pronounced growth peaks, whereas tested compounds showed a marked pronounced and progressive decline, especially with MB, which caused total inhibition from 96 h (Figure 3B).

These data corroborate preliminary results (Figure S1B_1_–B_4_), where treating promastigotes with different concentrations of MB (3.13–200 μM) showed a significant reduction (*p* < 0.05) at 48 h at higher concentrations (from 50 μM) and total viability loss from 12.50 μM at 72 h. The growth profiles of NMB, NMB-B, and NMB-P are like the reference drug (Figure 3C,E). A significant reduction (*p* < 0.05) in proliferation from 24 h was observed in MB from 48 h with NMB-B; from 72 h with NMB; and 96 h with NMB-P compared to MT. At 144 h, NMB-B showed a highly significant effect (*p* < 0.0001), while MB maintained this efficacy between 96 and 168 h.

### 3.2. Cytotoxicity on Different Mammalian Cell Types: Selective Efficacy of Repositioned and Novel Compounds

**Figure 4:**
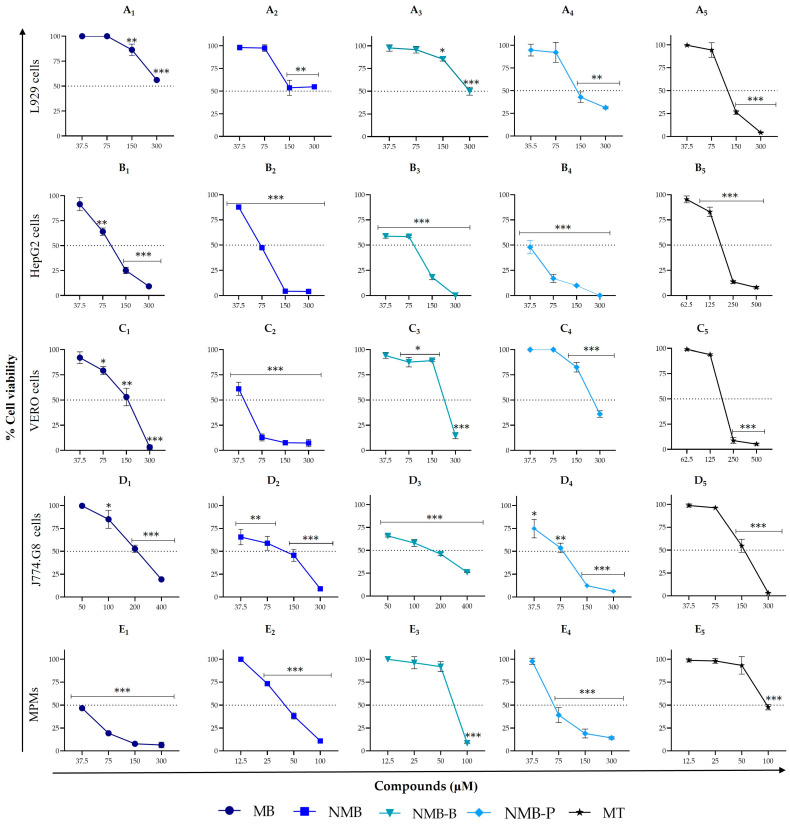
In vitro cytotoxic activity of methylene blue (MB), new methylene blue (NMB), new methylene blue B (NMB-B), new methylene blue P (NMB-P), and miltefosine (MT) on mammalian cells after 24 h of incubation. Panel A: L929 cells (0.5 × 10^5^/well) treated with MB (**A_1_**), NMB (**A_2_**), NMB-B (**A_3_**), NMB-P (**A_4_**), and MT (**A_5_**). Panel B: HepG2 cells (0.5 × 10^5^/well) treated with MB (**B_1_**), NMB (**B_2_**), NMB-B (**B_3_**), NMB-P (**B_4_**), and MT (**B_5_**). Panel C: VERO cells (10^5^/well) treated with MB (**C_1_**), NMB (**C_2_**), NMB-B (**C_3_**), NMB-P (**C_4_**), and MT (**C_5_**). Panel D: J774.G8 cells (10^5^/well) treated with MB (**D_1_**), NMB (**D_2_**), NMB-B (**D_3_**), NMB-P (**D_4_**), and MT (**D_5_**). Panel E: Murine peritoneal macrophages—MPMs (3 × 10^5^/well)—treated with MB (**E_1_**), NMB (**E_2_**), NMB-B (**E_3_**), NMB-P (**E_4_**), and MT (**E_5_**). Data represent mean ± SD. (*) *p* < 0.05; (**) *p* < 0.001; (***) *p* < 0.0001 when compared to positive control (untreated cells) by one-way ANOVA and Dunnett’s post-test.

The cytotoxicity of MB, NMB, NMB-B, and NMB-P was evaluated in five different types of mammalian host cells, four cell lines (L929, HepG2, VERO, and J774.G8), and one primary cell type (MPMs). The compound’s toxicity was compared with MT, a widely known leishmanicidal drug. Dose–response curves (Figure 4) demonstrated that the reduction in viability was dose-dependent in all treatments. MB, at lower concentrations (37–100 μM), resulted in viability > 50% (Figure 4A_1_,B_1_,C_1_,D_1_), except on MPMs. Both NMB and NMB-B maintained this viability pattern in L929 cells (Figure 4A_2_,A_3_). MT, at concentrations ≥ 300 μM, eliminated the viability of VERO cells (Figure 4C_5_), whereas NMB-P was less toxic to these cells (Figure 4C_4_). The ruthenium complexes exhibited similar action to MT on MPMs, with viability > 50% at exposures up to 70 μM (Figure 4E_3_–E_5_).

Subsequently, the CC_50_ and respective SI for promastigote forms (SI^P^) and ex vivo amastigotes (SI^A^) were determined (Table 1). The compounds exhibited low to moderate or no toxicity, with CC_50_ values up to 521 times higher than the IC_50_ observed for *L. amazonensis*. Among the 40 conditions analyzed, 45% exhibited SI between 10 and 50, 15% between 50 and 100, and 13% exceeded 100. MB showed the best activity on L929 cells (CC_50_ = 321.65 ± 3.74 μM, SI^P^ = 5.24 and SI^A^ = 20.55), as did NMB (CC_50_ = 280.25 ± 2.19 μM) with an SI^A^ close to that of MT (SI^A NMB^ = 105.35 and SI^A MT^ = 116.20). The ruthenium complexes were less toxic than the reference drug on L929 and VERO cells.

Concerning macrophages (J774.G8 and MPMs), which are targeted host cells of *Leishmania* infection, J774.G8 cells were less susceptible than MPMs, including treatment with MT. However, regarding selectivity, the SI^P^ and SI^A^ with NMB-P were higher in MPMs than in J774.G8. In contrast, with MT, the SI values for both parasite forms were lower in MPMs compared to lineage-derived macrophages.

### 3.3. Leishmania amazonensis ΔΨm Reduction

**Figure 5:**
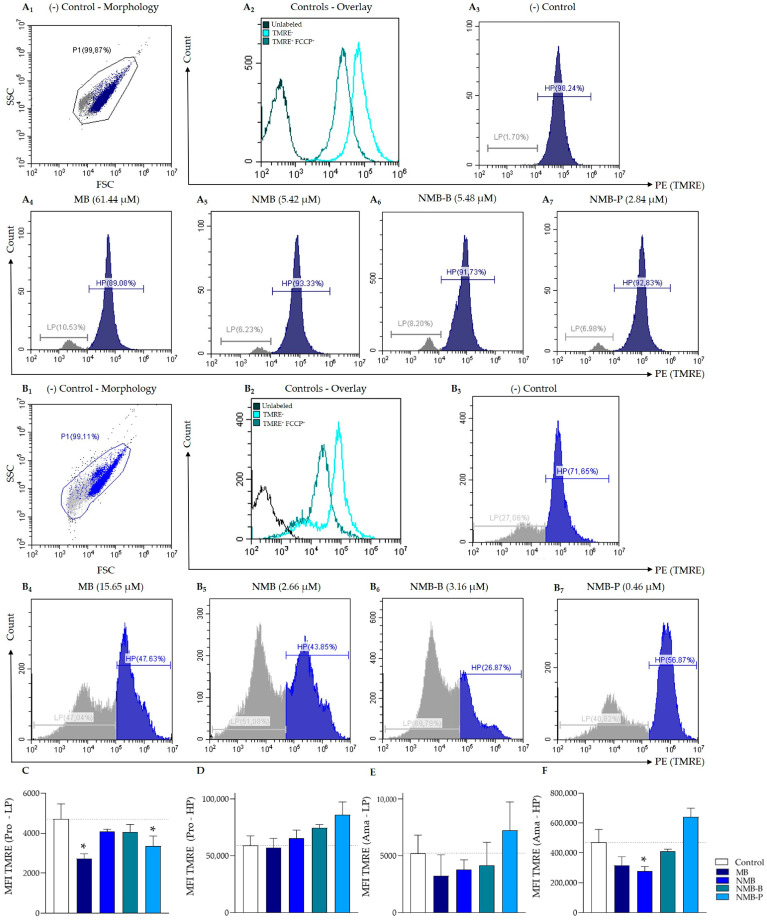
Mitochondrial membrane potential (ΔΨm) evaluation by flow cytometry on *Leishmania amazonensis* (5 × 10^6^ parasites/mL) exposed to IC_50_ of methylene blue (MB), new methylene blue (NMB), new methylene blue B (NMB-B), and new methylene blue P (NMB-P) for 24 h. Panel A (**A_1_**–**A_7_**): evaluation on promastigote forms (*L. amazonensis* PH8 strain). Panel B (**B_1_**–**B_7_**): evaluation on ex vivo amastigote forms (*L. amazonensis* LTB0016 strain). (**A_1_**,**B_1_**) Untreated TMRE^+^ parasites (negative control). (**A_2_**,**B_2_**) Untreated TMRE^+^ and FCCP^+^ parasites (positive control). (**A_3_**,**B_3_**) = TMRE fluorescence intensity of negative control. (**A_4_**–**A_7_**,**B_4_**–**B_7_**) TMRE fluorescence intensity of parasites treated with the IC_5__0_ of MB (**A_4_**,**B_4_**), NMB (**A_5_**,**B_5_**), NMB-B (**A_6_**,**B_6_**), and NMB-P (**A_7_**,**B_7_**). (**C**,**D**) MFI for promastigotes in LP (**C**) and HP (**D**) populations. (**E**,**F**) MFI for ex vivo amastigotes in LP (**E**) and HP (**F**) populations. Data represent mean ± SD. (*) *p* < 0.05 when compared to negative control by one-way ANOVA and Dunnett’s post-test. SSC = Side Scatter (granularity); FSC = Forward Scatter (relative size); PE = Channel; TMRE = Tetramethyrhodamine ethyl; FCCP = Carbonyl cyanide 4-(trifluoromethoxy)phenylhydrazone; LP = Low Population; HP = High Population; MFI = Median Fluorescence Intensity; IC_50_ = 50% Maximal Inhibitory Concentration.

Flow cytometry was employed on parasites exposed for 24 h to the IC_50_ of the compounds to evaluate the possible mechanisms of action. Initially, ΔΨm was assessed using TMRE labeling in promastigote (Figure 5A_1_–A_7_) and ex vivo amastigote forms (Figure 5B_1_–B_7_) of *L. amazonensis*. Morphology dot plots (SSC × FSC—Figure 5A_1_,B_1_) showed two distinct populations based on relative cell size and granularity, defined as parasites with lower (LP—Low Population) and higher viability (HP—High Population), presented in gray and blue, respectively (Figure 5). This profile was confirmed by the AV/PI viability assay (Figure S4). Untreated parasites exhibited higher fluorescence when incubated with TMRE alone than those coincubated with FCCP (overlays in Figure 5A_2_,B_2_). Furthermore, controls showed high enrichment in HP (promastigotes = 94.73 ± 3.37% and amastigotes = 67.48 ± 8.50%—Figure 5A_3_,B_3_), whereas treated parasites exhibited reductions (up to 25%) in this subpopulation (Figure 5A_4_–A_7_,B_4_–B_7_).

The VI_ΔΨm_ are presented in Table 2. In LP promastigotes, the compounds, particularly MB and NMB-P (*p* < 0.05—Figure 5C), reduced 13% to 41%. Mitochondrial damage was greater in amastigotes, except for the treatment with NMB-P. In LP ex vivo amastigotes, the loss of potential ranged from 27% to 37%, while in HP, it was higher, varying from 43% to 61%. Considering both subpopulations, the data in amastigotes are notable, with decreases of 98%, 90%, and 76% after treatment with MB, NMB-B, and NMB, respectively. Therefore, these results indicate that the compounds significantly impact the ΔΨm of *L. amazonensis* in a manner dependent on the compound and parasitic form.

### 3.4. ROS Production Triggering

**Figure 6:**
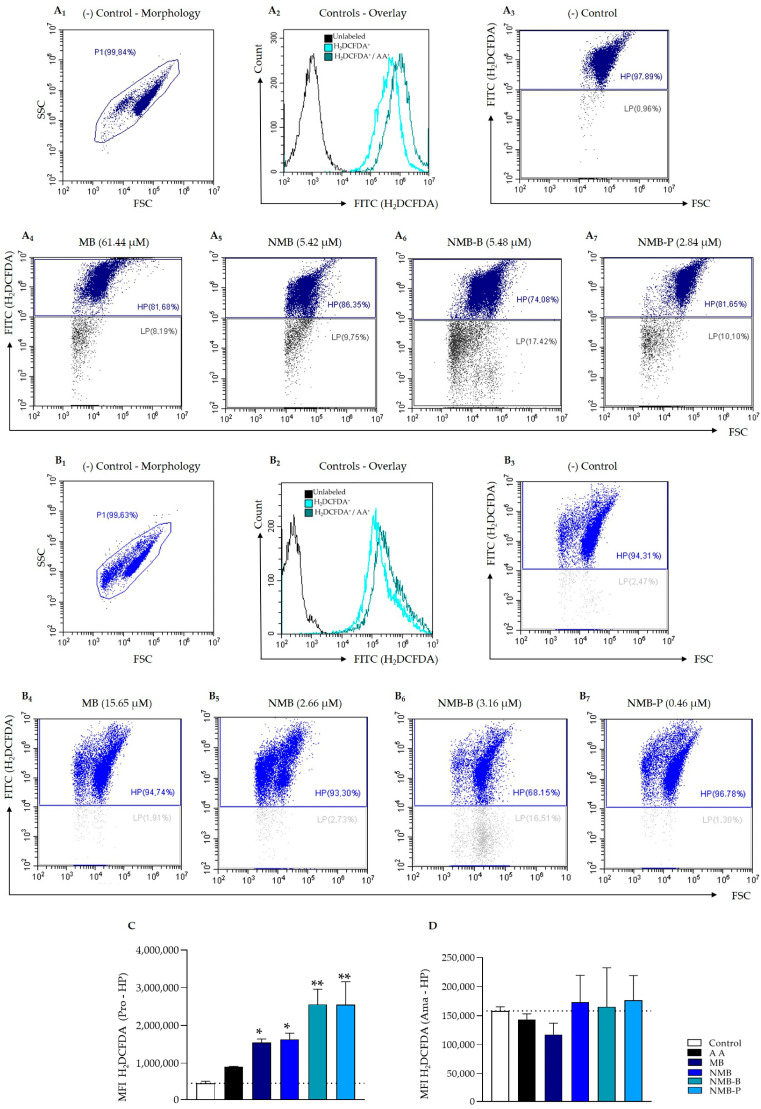
Reactive oxygen species (ROS) production evaluation by flow cytometry on *Leishmania amazonensis* (5 × 10^6^ parasites/mL) exposed to IC_50_ of methylene blue (MB), new methylene blue (NMB), new methylene blue B (NMB-B), and new methylene blue P (NMB-P) for 24 h. Panel A (**A_1_**–**A_7_**): evaluation on promastigote forms (*L. amazonensis* PH8 strain). Panel B (**B_1_**–**B_7_**): evaluation on ex vivo amastigote forms (*L. amazonensis* LTB0016 strain). (**A_1_**,**B_1_**) Untreated H_2_DCFDA^+^ parasites (negative control). (**A_2_**,**B_2_**) Untreated H_2_DCFDA^+^ and AA^+^ parasites (positive control for ROS production). (**A_3_**,**B_3_**) = H_2_DCFDA fluorescence intensity of negative control. (**A_4_**–**A_7_**,**B_4_**–**B_7_**) H_2_DCFDA fluorescence intensity of parasites treated with the IC_5__0_ of MB (**A_4_**,**B_4_**), NMB (**A_5_**,**B_5_**), NMB-B (**A_6_**,**B_6_**), and NMB-P (**A_7_**,**B_7_**). MFI in promastigotes HP H_2_DCFDA^+^ (**C**) and ex vivo amastigotes (**D**) are shown. Data represent mean ± SD. (*) *p* < 0.05; (**) *p* < 0.001 when compared to negative control by one-way ANOVA and Dunnett’s post-test. SSC = Side Scatter (granularity); FSC = Forward Scatter (relative size); FITC = Channel; H_2_DCFDA = 2′,7′-dichlorodihydrofluorescein diacetate; AA = Antimycin A; LP = Low Population; HP = High Population; Pro = Promastigotes; Ama = ex vivo Amastigotes; IC_50_ = 50% Maximal Inhibitory Concentration.

In the analysis of ROS generation using the H_2_DCFDA probe, AA was used as an inducer and control for the technique (overlays in Figure 6A_2_,B_2_). Untreated promastigotes exhibited fluorescence predominantly in the HP region, with 97.23 ± 2.44% of events (Figure 6A_3_). In contrast, treated parasites showed enrichment in the LP region, ranging from 7.00 ± 0.86% to 14.32 ± 3.10% (Figure 6A_4_–A_7_), indicating cellular damage. This profile was not observed in ex vivo amastigotes, where the percentages were similar to the control (Figure 6B_3_–B_7_). In this parasite form, the VI_ROS_ of all compounds were similar to AA in both LP and HP (Table 3—Figure 6D). Conversely, in promastigotes, there was a remarkable increase in VI_ROS_. MB and NMB tripled the ROS generation (*p* < 0.05), while NMB-B and NMB-P enhanced it five-fold (*p* < 0.001), surpassing AA (VI_ROS_ = 2.07 ± 0.23—Figure 6C). In the LP region, MB and NMB exceeded AA (VI_ROS_ = 0.52 ± 0.13), while NMB-B and NMB-P doubled this value.

### 3.5. Cellular Death in Leishmania amazonensis: Potential Mechanisms from Early Apoptosis to Necrosis

**Figure 7:**
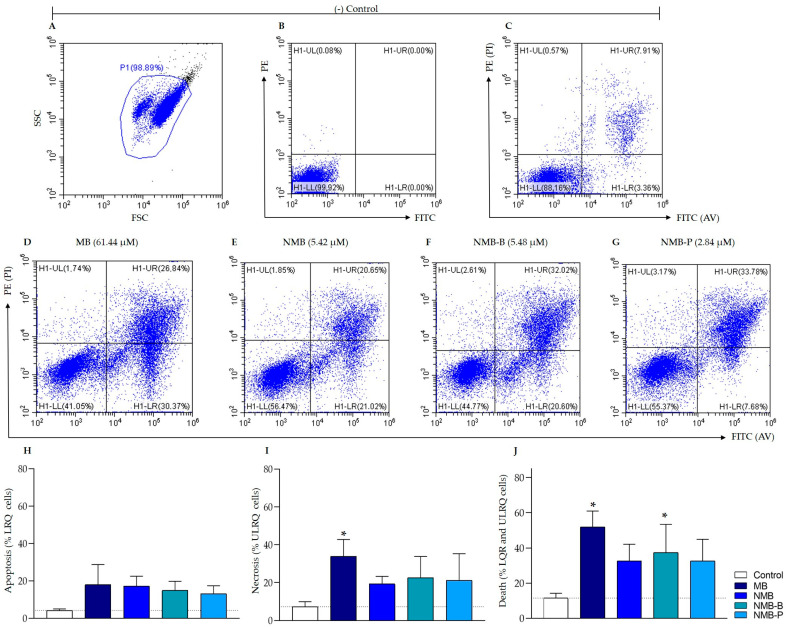
Cell death evaluation by flow cytometry on promastigote forms of *Leishmania amazonensis* PH8 strain (10^6^ parasites/mL) exposed to IC_50_ of methylene blue (MB), new methylene blue (NMB), new methylene blue B (NMB-B), and new methylene blue P (NMB-P) for 24 h and labeled with AV and PI (**D**–**G**). Untreated parasites were used as negative control (**A**–**C**). The percentage of apoptotic cells (**H**), necrotic cells (**I**), and general death (**J**) are shown. Data represent mean ± SD. (*) *p* < 0.05 when compared to negative control by one-way ANOVA and Dunnett’s post-test. SSC = Side Scatter (granularity); FSC = Forward Scatter (relative size); FITC and PE = Channels; AV = Annexin V; PI = Propidium Iodide; LQR = Low Right Quadrants; ULRQ = Upper Left and Right Quadrants; IC_50_ = 50% Maximal Inhibitory Concentration.

After 24 h of treatment with the IC_50_ of the compounds and labeling with AV/PI, mortality in promastigote forms ranged from 38.93 ± 8.06% to 57.10 ± 9.03% (Figure 7), confirming the inhibition data (Figure 2A_1_–A_5_). MB and NMB-B were notably significant (*p* < 0.05—Figure 7J). Approximately 12 ± 1.62% of events were AV^+^ and PI^−^, while 25 ± 6.37% were PI^+^, suggesting late apoptosis or necrosis. Preliminary assays showed a drastic reduction in viability from 99.81% to 12.89% with 200 μM of MB (Figure S5).

According to the analysis of subpopulations (Figure S4), the LP region showed more apoptotic and/or necrotic events compared to the HP region, reflecting the greater sensitivity of parasites in this area to the compounds. All compounds in the LP region exhibited significantly higher AV^+^ events (*p* < 0.05) than the control. Even in the more resistant HP region, there was a significant increase in AV^+^ events with MB and NMB-B (*p* < 0.05). Additionally, exposure to NMB-P in the HP region resulted in a significantly (*p* < 0.0001) higher number of necrotic events, consistent with its IC_50_ (Table 1).

**Figure 8:**
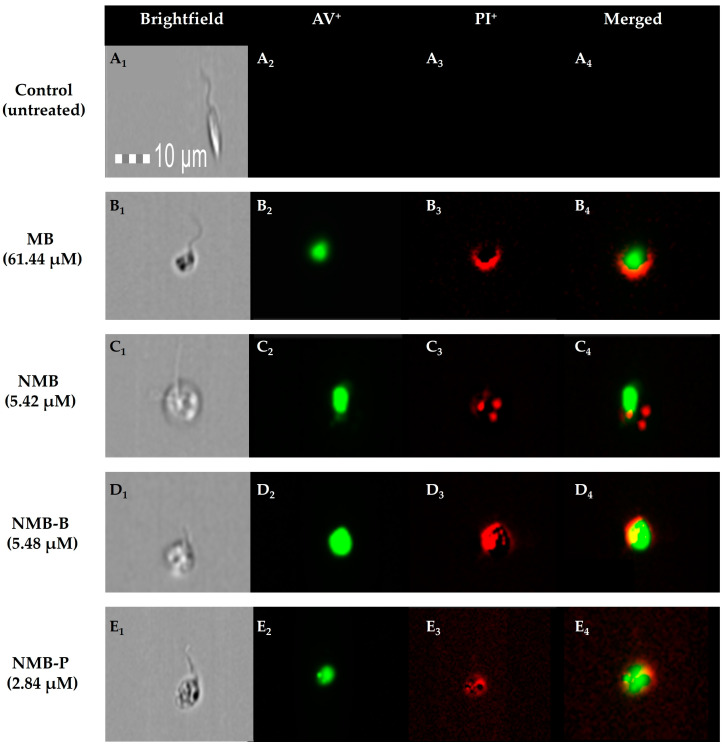
Cell death evaluation by imaging flow cytometry (Ammnis Stream MK XII) on promastigote forms of *Leishmania amazonensis* PH8 strain (10^6^ parasites/mL) exposed to IC_50_ of methylene blue (MB), new methylene blue (NMB), new methylene blue B (NMB-B), and new methylene blue P (NMB-P) for 24 h and labeled with AV and PI. Panel A (**A_1_**–**A_4_**): Negative control (untreated parasites) displayed in brightfield (**A_1_**), AV labeling (**A_2_**), PI labeling (**A_3_**), and merged (**A_4_**). Panel B (**B_1_**–**B_4_**): Parasites treated with MB displayed in brightfield (**B_1_**), AV labeling (**B_2_**), PI labeling (**B_3_**), and merged (**B_4_**). Panel C (**C_1_**–**C_4_**): Parasites treated with NMB displayed in brightfield (**C_1_**), AV labeling (**C_2_**), PI labeling (**C_3_**), and merged (**C_4_**). Panel D (**D_1_**–**D_4_**): Parasites treated with NMB-B displayed in brightfield (**D_1_**), AV labeling (**D_2_**), PI labeling (**D_3_**), and merged (**D_4_**). Panel E (**E_1_**–**E_4_**): Parasites treated with NMB-P displayed in brightfield (**E_1_**), AV labeling (**E_2_**), PI labeling (**E_3_**), and merged (**E_4_**). AV = Annexin V; PI = Propidium Iodide; IC_50_ = 50% Maximal Inhibitory Concentration.

**Figure 9:**
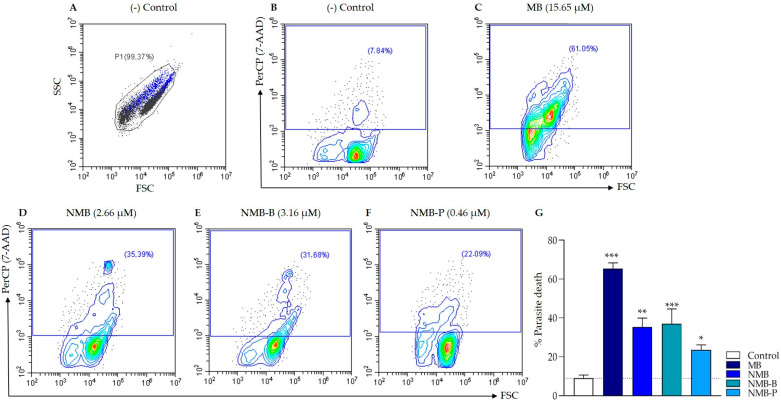
Cell death evaluation by flow cytometry on ex vivo amastigote forms of *Leishmania amazonensis* LTB0016 strain (10⁶ parasites/mL) exposed to the IC_5__0_ of methylene blue (MB), new methylene blue (NMB), new methylene blue B (NMB-B), and new methylene blue P (NMB-P) for 24 h and labeled with 7-AAD. (**A**,**B**): Negative control (untreated parasites) showing morphology (**A**) and 7-AAD labeling (**B**). (**C**–**F**): Parasites treated parasites with MB (**C**), NMB (**D**), NMB-B (**E**), NMB-P (**F**), and labeled with 7-AAD. (**G**): Percentage of parasite death for each treatment compared to negative control. Data represent mean ± SD. (*) *p* < 0.05; (**) *p* < 0.001; (***) *p* < 0.0001 when compared to negative control by one-way ANOVA and Dunnett’s post-test. SSC = Side Scatter (granularity); FSC = Forward Scatter (relative size); PerCP = Channel; 7-AAD = 7-amino-actinomycin D; IC_50_ = 50% Maximal Inhibitory Concentration.

Image cytometry provided insights into the cellular death processes (Figure 8). Untreated parasites (Figure 8A_1_) displayed elongated and flagellated morphology typical of promastigotes, with high refringence and intact membranes. The absence of AV and PI labeling confirms cellular integrity (Figure 8A_2_–A_4_). However, the treatments resulted in evident structural damage (Figure 8B_1_,C_1_,D_1_,E_1_), possibly associated with different types of cell death, such as autophagy, apoptosis, and necrosis. Observed damages included cell rounding, cytoplasmic degradation, reduced refringence, membrane damage, flagellum loss, and possible vacuolation. The green fluorescence indicating phosphatidylserine exposure suggests early apoptosis (Figure 8B_2_,C_2_,D_2_,E_2_), while red fluorescence (PI binding to DNA) indicated late apoptosis or necrosis, with possible DNA fragmentation (Figure 8C_3_,C_4_) and release of genetic material into the extracellular medium (Figure 9B_4_,D_4_,E_4_).

High phosphatidylserine exposure was detected in ex vivo amastigote controls with AV labeling (77.13%), making it unsuitable as a cell death marker (Figure S6C), consistent with findings by Balanco et al. [75] and Wanderley et al. [76]. This pattern was absent in promastigotes [77,78]. Image cytometry showed control parasites with typical oval shape, non-prominent flagellum, intact membranes, and high refringence (indicative of parasite viability) while being AV^+^ (Figure S6E). Consequently, 7-AAD staining was performed (Figure 9). All compounds induced significant cell death (*p* < 0.05), ranging from 23.55 ± 2.56% to 65.42 ± 2.92%, suggestive of late apoptosis/necrosis. MB demonstrated the highest mortality rate (Figure 9C) with strong significance (*p* < 0.0001), followed by NMB and NMB-B (Figure 9D,E).

Subpopulation analysis (Figure S7) revealed that approximately 75.02 ± 0.57% of events in controls were in the HP region, with only 6 ± 2.08% of parasites being 7-AAD^+^, indicating high viability. Treatment significantly increased positivity (*p* < 0.001) from 19.60 ± 1.39% to 82.74 ± 3.95%, indicating membrane disruption and necrotic cell death. In the LP region, which corresponds to 17.71 ± 1.51% to 33.51 ± 3.12% of total events, 7-AAD^+^ percentages ranged from 0.42 ± 0.17% to 2.24 ± 0.67%. This lower labeling in LP might be due to the higher susceptibility of ex vivo amastigote forms to treatment (Table 1) and the LP region representing parasites with lower viability. Thus, the low 7-AAD labeling might be associated with secondary necrosis, where extensive cellular damage reduces the availability of detectable genetic material.

## 4. Discussion

The pursuit of more selective and less toxic therapies for leishmaniasis, particularly those administered orally and designed to overcome the parasite’s adaptive strategies and drug resistance mechanisms, remains a significant obstacle. This challenge can be interpreted as a “coevolutionary arms race” considering the Red Queen hypothesis [79]. In addition, the high toxicity and costs have largely hindered chemotherapy. Thus, the discovery of new drugs for CL capable of overcoming current obstacles is an urgent demand.

Numerous research teams have focused on evaluating substances with activity against *L. amazonensis* over the last decade exploring the activity of natural products [80,81,82,83,84,85,86], novel chemotypes [87,88,89,90,91], and repurposed drugs [92], using 24-h kinetics with IC_50_ values expressed in µM, as conducted in this study. These works emphasize in vitro investigations of mechanisms of action in promastigotes targeting the membrane and mitochondria, alongside cytotoxicity and selectivity analyses in mammalian cells.

Our findings align with literature, particularly in exploring mechanisms targeting different parasite structures, and in cytotoxicity and selectivity assessments. Notably, IC_50_ values ≤ 5.00 µM were observed in promastigotes using NMB, NMB-B, and NMB-P, as well as submicromolar concentration in amastigotes, a highly relevant parasite form for treatment (Table 1). There is evidence suggesting sensitivity similarities between ex vivo and intracellular amastigotes, warranting further investigation. Present et al. [93] evaluated a tubercidin analog and MT in ex vivo and intracellular amastigotes, reporting a mere 0.15 µM difference for the analog and 1.66 µM for MT. Similarly, Santos et al. [69] observed approximately a 4 µM difference between these amastigote populations challenged with nitroaromatic compounds.

In vitro potency determination is important for screening drug candidates for infectious diseases and hit-to-lead profiles serve as guidelines. While reference values are established for visceral leishmaniasis (VL) [94,95], data for CL remain scarce despite its substantial impact [37]. Katsuno et al. [96] proposed a generic criterion for substance selection, suggesting an SI > 10 in mammalian cells like HepG2 or VERO. For VL, they recommend an IC_50_ < 10 µM and an SI > 100. Our data are consistent with these guidelines, with 75% of compounds tested in promastigotes and amastigotes achieving an IC_50_ < 10 µM. Additionally, upon testing the cytotoxicity of four compounds across two parasitic forms and five cell types (HepG2, Vero, L929, J774.G8, and MPMs), we obtained SI values > 10 in most conditions.

Among the various cells susceptible to *Leishmania* infection, macrophages play a critical role in differentiation, proliferation, and infection regulation [97,98,99,100]. Previous studies [81,82,83,84,85,86,87,88,89] reported the greater sensitivity of primary cells, corroborating our findings, including for MT (Table 1). NMB-P stood out with SI > 150 on ex vivo amastigotes for primary and lineage macrophages. Preliminary in vitro screening with mammalian cell lines, especially hepatic and renal models, is important for the early identification of promising leishmanicidal compounds, given the well-known toxicity associated with first-line medications. However, further studies are needed to assess hepato- and nephrotoxicity in complex systems. In leishmaniasis chemotherapy, these toxic effects are not exclusive to antimonials [31,101,102,103] but have also been reported for amphotericin B [104,105,106,107,108,109], paromomycin [110], pentamidine [111,112], and MT [113,114,115,116].

In the cytotoxicity analysis using HepG2 cells, a widely used hepatic tumor line including studies on compound metabolism [117,118], MB, and NMB exhibited low toxicity (CC_50_ > 70 μM). These results contrast with those of Hameed et al. [119], who evaluated 101 substances against parasites, with 64.35% targeting *Leishmania mexicana* and found a correlation between high activity in trypanosomatids and elevated cytotoxicity in HepG2. In our investigation, only NMB-B and NMB-P demonstrated moderate cytotoxicity (CC_50_ < 36 μM) at concentrations similar to Amphotericin B (35.24 μM), as reported by Araújo et al. [120].

In VERO cells, the compounds exhibit antileishmanial activity at non-cytotoxic concentrations, a critical criterion for advancing drug candidates [121]. NMB-B and NMB-P demonstrated lower toxicity (CC_50_ > 230 μM) compared to MT (CC_50_ = 168.05 μM), with superior SI (Table 1). A previous study supports our findings, showing that phenothiazine possesses low or negligible cytotoxicity across various cell lines, underscoring their potential in leishmaniasis chemotherapy [122].

The role of non-canonical cells, such as fibroblasts, in leishmaniasis has been underestimated despite their ability to sustain parasite multiplication and survival, potentially contributing to persistence after self-healing or treatment [66]. The possibility of *Leishmania* remaining dormant in fibroblasts requires new therapeutic approaches. Cavalcante-Costa et al. [123] demonstrated that *L. amazonensis* infects fibroblasts through mechanisms distinct from phagocytosis, subverting cellular functions such as Ca^2+^ signaling and lysosomal exocytosis. Recently, Yektaeian et al. [124] revealed the potential for parasite detection in fibroblasts using iron oxide nanoparticles. Given their therapeutic importance, leishmanicidal substances have been evaluated for toxicity in hFB [125], 3T3 [126,127,128], and L929 [129,130,131] fibroblasts. In this study, the compounds showed no significant toxicity in L929 cells (CC_50_ > 150 µM).

Regarding the tested compounds, for MB without light treatment, the IC_50_ for promastigotes was reported as 100 μM [60], with no cytotoxicity observed in fibroblasts and macrophages [132], similar to our results for both MB and NMB on L929 and J744.G8 (Table 1). When combined with PDT, MB shows efficacy against species causing CL, promoting healing and reducing parasite burden in vivo [58,133,134,135] and in vitro, including against MT-resistant strains, with damage associated with ROS induction and ΔΨm loss [56,136]. Cabral et al. [136] reported a 2-fold increase in ROS levels and a 60% reduction in ΔΨm in *L. amazonensis* after MB treatment with PDT.

Aureliano et al. [57] demonstrated that PDT enhances MB activity, leading to mitochondrial damage observed through electron microscopy, an approach that may be instrumental in the elucidation of the antiparasitic agent mechanism of action [137]. However, the electron micrographs revealed vacuoles containing electron-dense material, which were not explored in the study and had been previously described [138,139]. These vacuoles may correspond to empty acidocalcisomes, previously reported in *Leishmania major* as compartments related to lysosomes, originating from multivesicular bodies [140]. Moreover, the authors did not discuss the presence of necrotic cells and the marked induction of autophagy, a process extensively studied [141], including *Leishmania* [142].

We present similar findings, even without PDT, where both MB and NMB increased ROS generation up to 5-fold (Table 3) and reduced ΔΨm by up to 61% (Table 2). These mechanisms are linked to the high mortality observed in promastigotes (Figure 7) and ex vivo amastigotes (Figure 9) following treatment with the compounds’ IC_50_. Significant changes in mitochondrial activity and cell morphology, including cell body rounding, were also reported in *L. major* and *Leishmania braziliensis* under PDT with MB by Pinto et al. [52], as documented through image cytometry for MB, NMB, and its complexes (Figure 8B_1_,C_1_,D_1_,E_1_).

Ruthenium complexes (RCs) have demonstrated potent antileishmanial activity in vitro, achieving inhibitory concentrations at low to submicromolar doses [64,143]. Costa et al. [63] observed IC_50_ values ranging from 0.52 to 7.52 µM in *L. amazonensis* promastigotes, though with cytotoxicity in Raw 264.7 macrophages (CC_50_ between 2.14 and 8.73 µM). Our data confirm the efficacy of RCs with NMB-B (IC_50pro_ = 5.48 µM; IC_50ama_ = 3.16 µM) and NMB-P (IC_50pro_ = 2.84 µM; IC_50ama_ = 0.46 µM), as well as their low cytotoxicity in J774.G8 macrophages (CC_50_ > 65 µM) and high selectivity against both parasite forms (SI > 10). These results are in agreement with Fandzloch et al. [144], who reported CC_50_ > 70 µM on J774.2 cells and > 58 µM on VERO cells. In these cell types, we observed CC_50_ > 238 µM and SI ranging from 45 to 525.30. In vivo, RCs significantly reduced lesion size and parasite load [65,145].

One of the mechanisms of action of RCs involves the release of nitric oxide (NO), which plays a crucial role in controlling *Leishmania* infections by inducing apoptotic-like death in intracellular amastigotes [146,147]. In promastigotes, RCs significantly reduced proliferation, while in infected macrophages, parasitic death was induced by increased NO production, concomitant with elevated expression of Akt, NF-kB, and iNOS in the macrophages [148]. In environments with ROS, NO can react to form more reactive and toxic species [149].

Costa et al. [150] suggest that RCs act as oxidizing agents, inducing *L. amazonensis* death through an apoptosis-like process characterized by increased ROS production and ΔΨm depolarization, consistent with our findings (Table 2 and Table 3). Additionally, similar morphological alterations were observed, such as cell rounding, mitochondrial compromise, and DNA fragmentation, also described in *L. major* [62], suggesting a cell death profile through a mechanism resembling late apoptosis/necrosis, as demonstrated by the markers PI and 7-AAD (Figure 7, Figure 8 and Figure 9).

## 5. Conclusions

Overall, the compounds demonstrated promising leishmanicidal activity against both stages of *L. amazonensis*, achieving submicromolar concentration and showing selectivity toward renal cells, hepatocytes, fibroblasts, and macrophages, thereby confirming our initial hypothesis. Additionally, the substances exhibited early/late apoptosis features, such as phosphatidylserine externalization, mitochondrial membrane potential loss, morphological changes, and DNA fragmentation. These effects support the conclusion that repurposed compounds and novel ruthenium-complexed chemotypes are potential candidates for antileishmanial drugs. This encourages the pursuit of further assays to maximize leishmanicidal effects while reducing cytotoxicity, aiming for a synergistic therapeutic approach.

## Figures and Tables

**Table 1:** In vitro activities of methylene blue (MB), new methylene blue (NMB), new methylene blue B (NMB-B), new methylene blue P (NMB-P), and miltefosine on promastigote forms of *Leishmania amazonensis* PH8 strain (10^6^ parasites/mL), ex vivo amastigote forms of *L. amazonensis* LTB0016 strain (10^6^ parasites/mL), mammalian cells [L929, HepG2 (0.5 × 10^5^ cells/well), VERO, J774.G8 (10^5^ cells/well), and murine peritoneal macrophages—MPMs (3 × 10^5^ cells/well)]—and their respective selective indexes (SI) after 24 h of incubation.

Compounds	Antiparasitic Activity		Cytotoxicity/Selective Indexes				
	IC_50_ μM		CC_50_ μM/(SI^P^/SI^A^)				
	Pro.	Ama.	L929	HepG2	VERO	J744.G8	MPMs
MB	61.44 ± 4.41	15.65 ± 2.23	321.65 ± 3.74	95.96 ± 3.28	141.05 ± 5.58	206.20 ± 9.19	36.02 ± 3.69
			(5.24/20.55)	(1.56/6.13)	(2.30/9.01)	(3.36/13.18)	(0.59/2.30)
NMB	5.42 *** ± 0.81	2.66 ± 0.95	280.25 ± 2.19	71.56 ± 3.15	44.73 ± 3.40	68.50 ± 2.12	31.27 ± 5.72
			(51.71/105.35)	(13.20/26.90)	(8.25/16.82)	(12.64/25.75)	(5.77/11.76)
NMB-B	5.48 *** ± 1.73	3.16 ± 0.37	270.30 ± 8.48	34.24 ± 7.31	247.95 ± 32.88	129.80 ± 7.35	65.91 ± 1.69
			(49.32/85.54)	(6.25/10.84)	(45.25/78.47)	(23.69/41.08)	(12.03/20.86)
NMB-P	2.84 *** ± 0.80	0.46 ± 0.34	150.40 ± 12.02	35.05 ± 2.08	239.80 ± 27.57	69.82 ± 10.38	71.63 ± 1.85
			(52.96/326.96)	(12.34/76.20)	(84.44/521.30)	(24.58/151.78)	(25.22/155.72)
Miltefosine	23.20 ± 2.55	0.97 ± 0.40	112.71 ± 18.22	181.00 ± 11.31	168.05 ± 2.75	155.60 ± 8.48	102.93 ± 4.77
			(4.86/116.20)	(7.80/186.60)	(7.24/173.25)	(6.71/160.41)	(4.44/106.11)

Pro. = Promastigotes. Ama. = Amastigotes. IC_50_ = 50% Maximal Inhibitory Concentration. CC_50_ = 50% Maximal Cytotoxicity Concentration. Selectivity Index (SI) = CC_50/_IC_50_. SI^P^ = Selectivity Index on promastigote forms of *L. amazonensis* PH8 strain. SI^A^ = Selectivity Index on ex vivo amastigote forms of *L. amazonensis* LTB0016 strain. Data represent the mean ± SD. (***) *p* < 0.0001 when compared to the standard drug (MT) by one-way ANOVA and Dunnett’s post-test.

**Table 2:** Flow cytometry analysis of mitochondrial membrane potential (ΔΨm) on promastigote forms of *Leishmania amazonensis* PH8 strain (5 × 10^6^ parasites/mL) and ex vivo amastigote forms of *L. amazonensis* LTB0016 strain (5 × 10^6^ parasites/mL) exposed to IC_50_ of methylene blue (MB), new methylene blue (NMB), new methylene blue B (NMB-B), and new methylene blue P (NMB-P) for 24 h and labeled with TMRE.

Compounds	IC_50_ (µM)	Promastigotes				IC_50_ (µM)	ex vivo Amastigotes			
		Low Population		High Population			Low Population		High Population	
		MFI	VI_ΔΨm_	MFI	VI_ΔΨm_		MFI	VI_ΔΨm_	MFI	VI_ΔΨm_
MB	61.44	2737.60 *	−0.41 ± 0.05	56,878.47	0.02 ± 0.03	15.65	3237.43	−0.37 ± 0.32	317,106.50	−0.61 ± 0.18
NMB	5.42	4095.05	−0.14 ± 0.20	65,590.87	0.11 ± 0.08	2.66	3770.37	−0.27 ± 0.06	277,557.80 *	−0.43 ± 0.20
NMB-B	5.48	4070.50	−0.13 ± 0.09	74,562.13	0.27 ± 0.16	3.16	4174.80	−0.31 ± 0.03	410,112.30	−0.59 ± 0.21
NMB-P	2.84	3359.03 *	−0.28 ± 0.03	86,029.23	0.49 ± 0.36	0.46	7215.47	0.21 ± 0.04	642,051.43	0.39 ± 0.13
Control	-	4702.03	0.00	59,161.87	0.00	-	5226.50	0.00	469,563.43	0.00

TMRE = Tetramethyrhodamine ethyl. IC_50_ = 50% Maximal Inhibitory Concentration. MFI = Median Fluorescence Intensity. VI_ΔΨm_ (Variation Index of Mitochondrial Membrane Potential) = (MT − MC)/MC, where MT corresponds to the TMRE MFI of treated parasites and MC corresponds to the TMRE MFI of control parasites. The control corresponds to untreated parasites labeled with TMRE. Data represent mean ± SD. (*) *p* < 0.05 when compared to control by one-way ANOVA and Dunnett’s post-test.

**Table 3:** Flow cytometry analysis of reactive oxygen species (ROS) production on promastigote forms of *Leishmania amazonensis* PH8 strain (10^6^ parasites/mL) and ex vivo amastigote forms of *L. amazonensis* LTB0016 strain (10^6^ parasites/mL) exposed to IC_50_ of methylene blue (MB), new methylene blue (NMB), new methylene blue B (NMB-B), and new methylene blue P (NMB-P) for 24 h and labeled with H_2_DCFDA.

Compounds	IC_50_ (µM)	Promastigotes				IC_50_ (µM)	Ex Vivo Amastigotes			
		Low Population		High Population			Low Population		High Population	
		MFI	VI_ROS_	MFI	VI_ROS_		MFI	VI_ROS_	MFI	VI_ROS_
MB	61.44	26,534.03	0.74 ± 0.12	1,658,186.80 *	3.56 ± 0.50	15.65	3151.50	1.28 ± 0.46	140,636.20	0.90 ± 0.29
NMB	5.42	31,489.80	0.87 ± 0.12	1,512,521.00 *	3.61 ± 0.08	2.66	2940.57	1.62 ± 0.90	131,669.43	0.84 ± 0.51
NMB-B	5.48	39,191.97	1.06 ± 0.11	2,639,177.00 **	5.81 ± 0.64	3.16	2116.87	1.11 ± 0.48	131,896.27	0.85 ± 0.49
NMB-P	2.84	34,607.80	1.02 ± 0.48	2,325,902.50 **	5.39 ± 1.83	0.46	3063.17	1.27 ± 0.45	140,291.57	0.90 ± 0.45
AA	10	19,775.40	0.52 ± 0.13	890,705.80	2.07 ± 0.23	10	3011.23	1.14 ± 0.38	143,227.25	0.60 ± 0.53
Control	-	39,868.50	-	452,909.35	-	-	2436.40	-	158,240.00	-

H_2_DCFDA = 2′,7′-dichlorodihydrofluorescein diacetate. IC_50_ = 50% Maximal Inhibitory Concentration. AA = Antimycin A. MFI = Median Fluorescence Intensity. VI_ROS_ (Variation Index of ROS production) = MT/MC, where MT corresponds to the H_2_DCFDA MFI of treated parasites, and MC corresponds to the H_2_DCFDA MFI of control parasites. The control corresponds to untreated parasites labeled with H_2_DCFDA. Data represent mean ± SD. (*) *p* < 0.05; (**) *p* < 0.001 when compared to negative control by one-way ANOVA and Dunnett’s post-test.

## Data Availability

Data are contained within the article and Supplementary Materials.

## Supplementary Materials

The following supporting information can be downloaded at https://www.mdpi.com/article/10.3390/biology14121709/s1. Figure S1: Growth curve and assessment of MB concentrations on promastigote forms of *L. amazonensis* in vitro for 72 h; Figure S2: Flow cytometric evaluation of fluorescence on promastigote forms of *L. amazonensis*; Figure S3: Flow cytometric evaluation of cell death markers on promastigote forms of *L. amazonensis*; Figure S4: Cell death evaluation by flow cytometry on subpopulations of *L. amazonensis* promastigotes exposed to IC_50_ of the compounds for 24 h; Figure S5: Cell death evaluation by flow cytometry on promastigote forms of *L. amazonensis* exposed to 200 μM of MB for 24 h; Figure S6: Phosphatidylserine exposure in ex vivo amastigote forms of *L. amazonensis*; Figure S7: Cell death evaluation by flow cytometry on subpopulations of *L. amazonensis* ex vivo amastigote forms exposed to IC_50_ of the compounds for 24 h.
