# Supplementary material for: From Dyes to Drugs? Selective Leishmanicidal Efficacy of Repositioned Methylene Blue and Its Derivatives in In Vitro Evaluation
Source: Biology (Basel). 2025 Nov 30;14(12):1709. doi: 10.3390/biology14121709 (PMC12730333; doi:10.3390/biology14121709)
Supplement: Supplementary file 1 [file biology-14-01709-s001.zip › biology-3347590-supplementary.pdf]

biology

MDPI

Article

From Dyes to Drugs? Selective Leishmanicidal Efficacy of Repositioned Methylene Blue and Its Derivatives in an In Vitro Evaluation

Deyvison Rhuan Vasco-dos-Santos, Juliana Almeida-Silva, Ludmila Ferreira de Almeida Fiuza, Natalia Vacani-Martins, Zênis Novais da Rocha, Maria de Nazaré Correia Soeiro, Andrea Henriques-Pons, Eduardo Caio Torres-Santos and Marcos André Vannier-Santos

Supplementary Materials

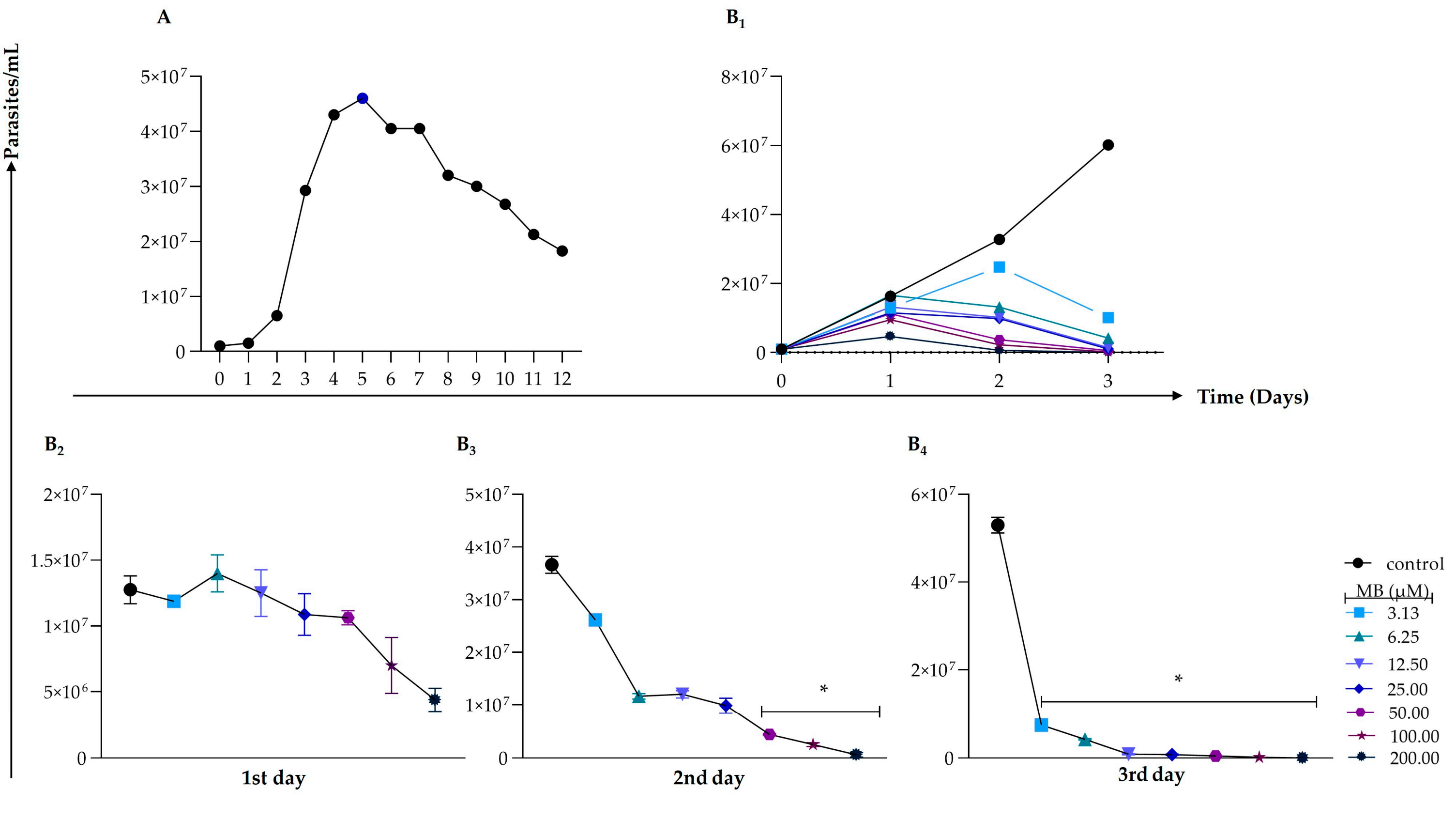

Figure S1. Growth curve (A) and assessment of methylene blue (MB) concentrations (3.13 - 200 µM) on promastigote forms *of Leishmania amazonensis* PH8 strain (10^6 parasites/mL) in vitro for 72 h (B_1). Evaluation of parasite proliferation after 24 h (B_2), 48 h (B_3), and 72 h (B_4) are shown. Viability was assessed by counting in a Neubauer chamber. Data represents mean ± SD. (*) $p < 0.05$ when compared to negative control by ANOVA and Tukey's post-test.

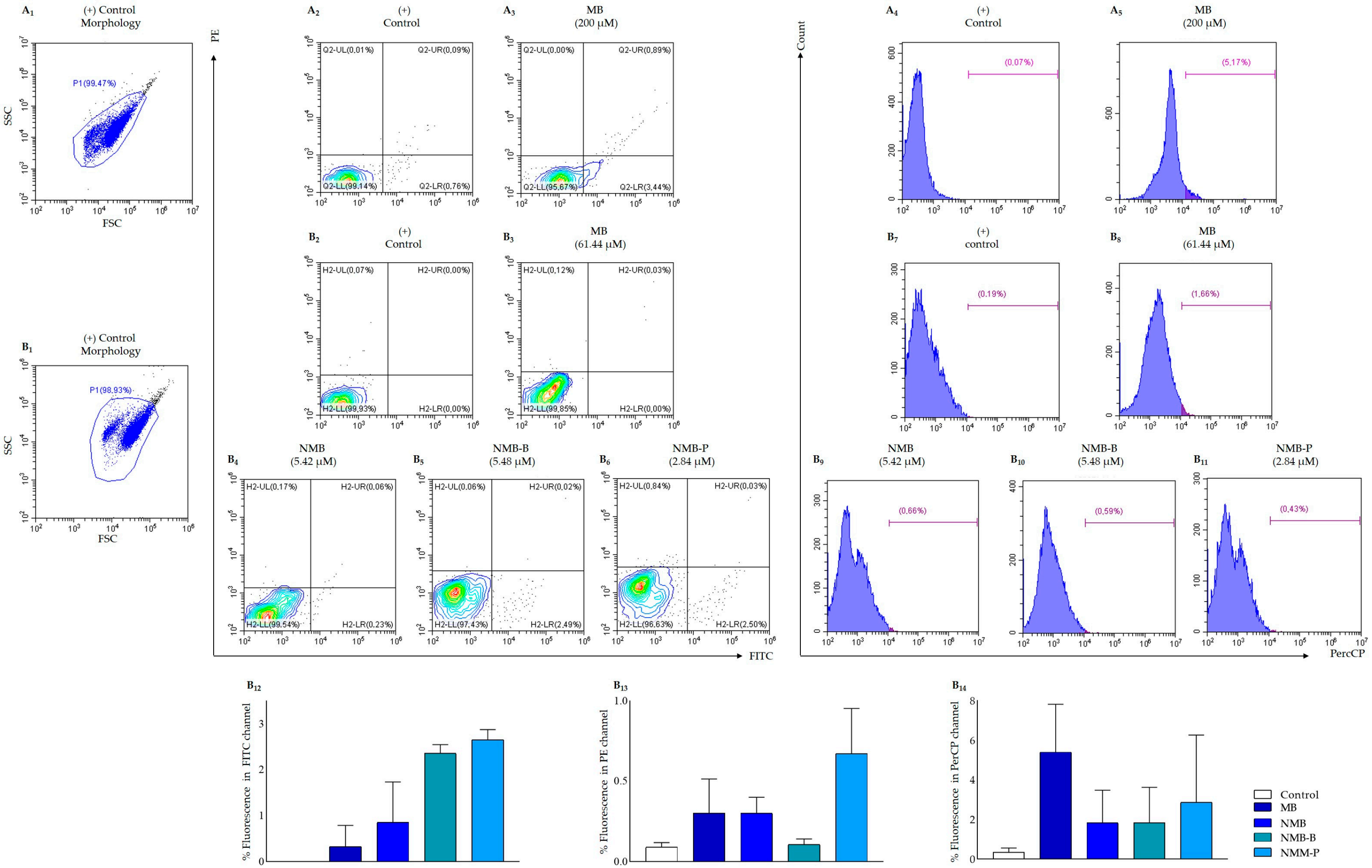

Figure S2. Flow cytometric evaluation of fluorescence on promastigote forms of *Leishmania amazonensis* PH8 strain (10^6 parasites/mL). Panel A: parasites exposed to 200 μM methylene blue (MB, A1-A5). Panel B: Parasites exposed to the IC_{50} of MB, new methylene blue (NMB), new methylene blue B (NMB-B), and new methylene blue P (NMB-P) after 24 h of incubation. The percentage of fluorescence in FITC (B_{12}), PE (B_{13}), and PerCP (B_{14}) are shown. Untreated live parasites were used as positive control. Data represents the mean ± SD. SSC = Side Scatter (granularity); FSC = Forward Scatter (relative size); FITC, PE and PerCP = Channels; IC_{50} = 50% maximal Inhibitory Concentration.

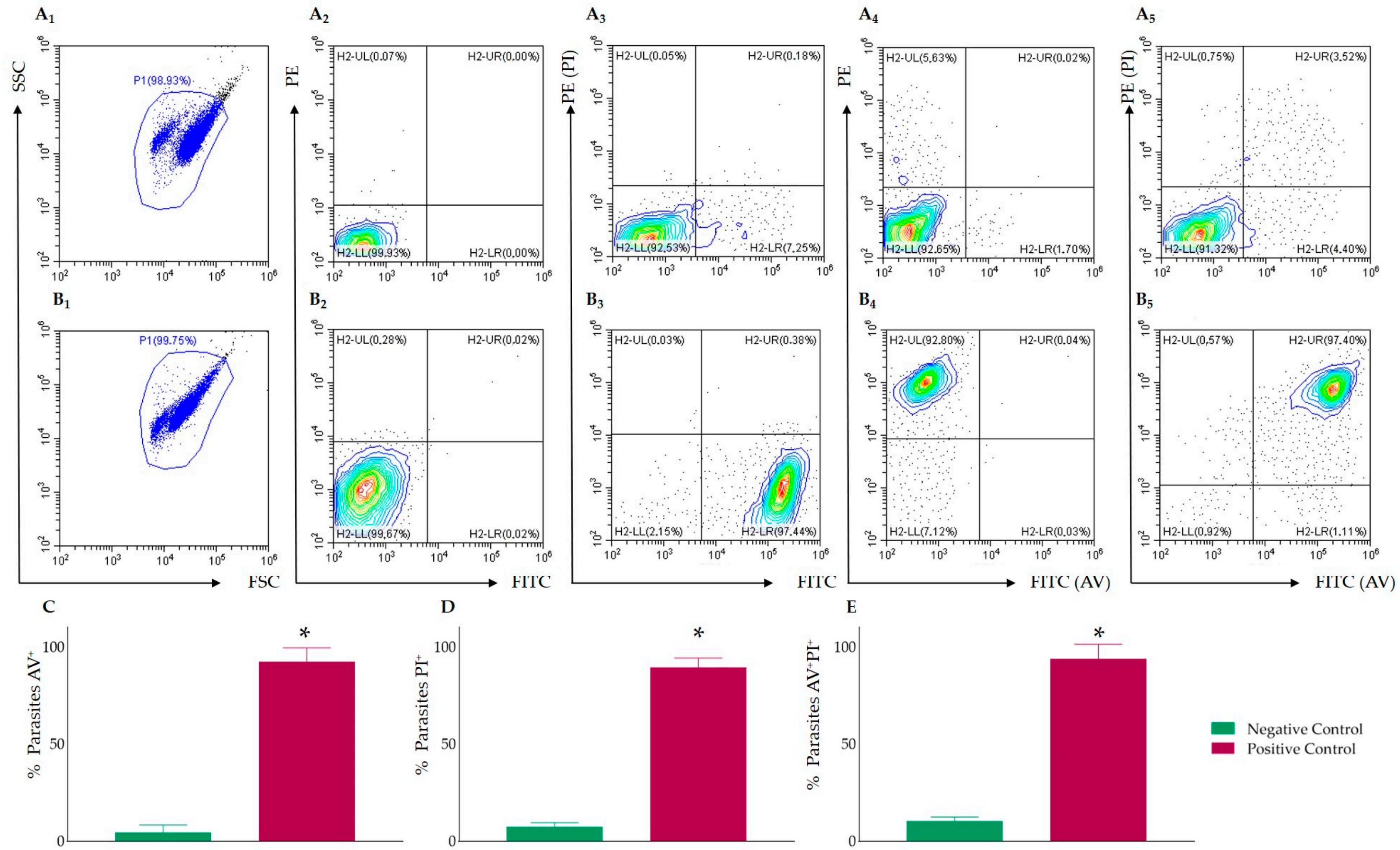

Figure S3. Flow cytometric evaluation of cell death markers (AV/PI) on promastigote forms of *Leishmania amazonensis* PH8 strain (10^6 parasites/mL). Panel A represents the negative control (untreated parasites, unlabeled or labeled). Panel B represents the positive control (killed by heating at 60 °C for 10 min, unlabeled or labeled). The percentage of cells AV^+ (C), PI^+ (D), and general death (E) are shown. Data represents mean ± SD. (*) $p < 0.05$ when compared to negative control by one-way ANOVA and Dunnett's post-test. SSC = Side Scatter (granularity); FSC = Forward Scatter (relative size); PE and FITC = Channels; AV = Annexin V; PI = Propidium Iodide.

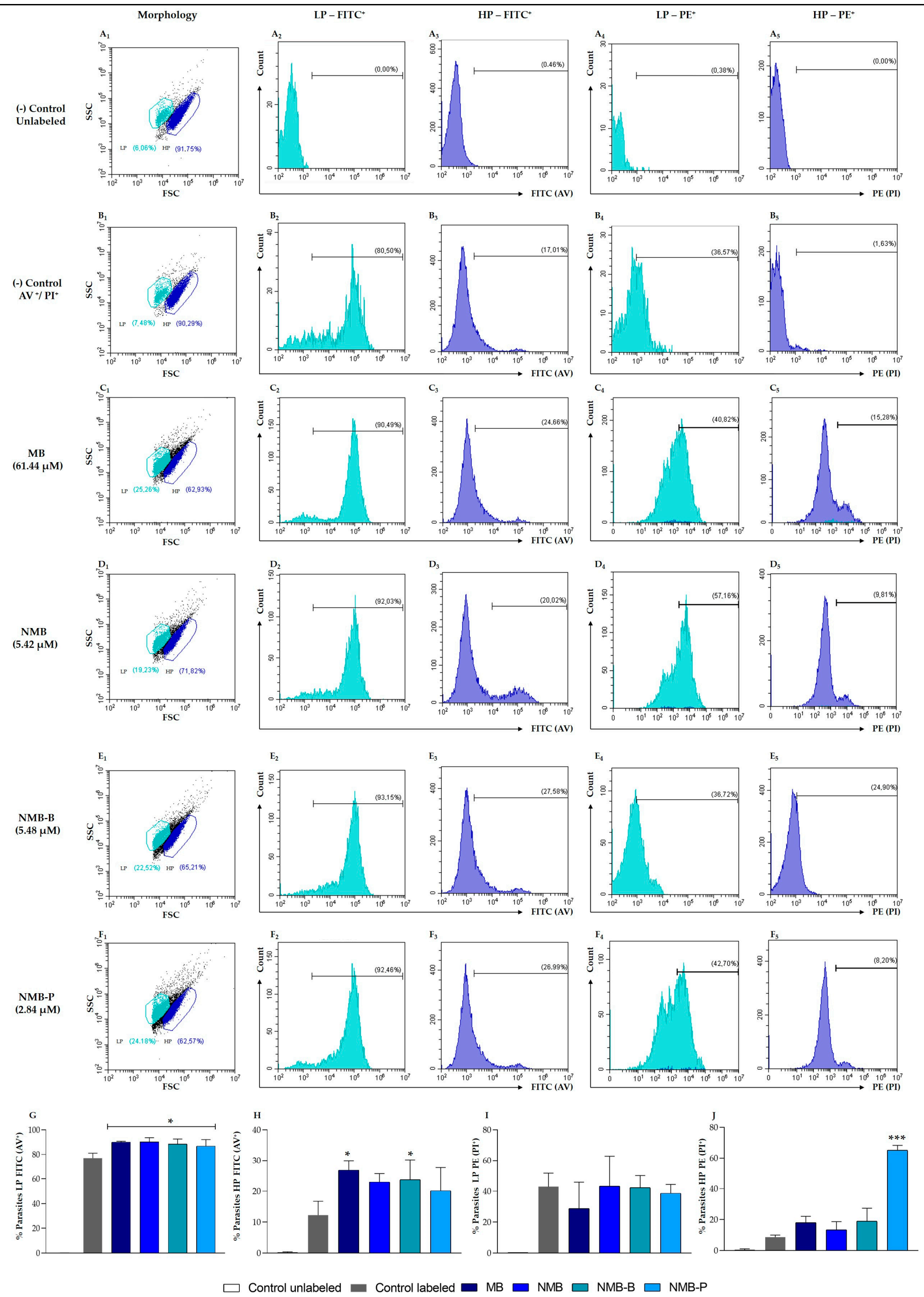

Figure S4. Cell death evaluation by flow cytometry on subpopulations of *Leishmania amazonensis* promastigotes (10^6 parasites/mL, PH8 strain) exposed to IC_{50} of methylene blue (MB – Panel C), new methylene blue (NMB – Panel D), new methylene blue B (NMB-B – Panel E), and new methylene blue P (NMB-P – Panel F) for 24 h. Samples were labeled with AV and PI. Untreated parasites were used as negative controls (Panels A and B). Dot plots illustrate morphological characteristics, while histograms represent retrogates within each subpopulation. The percentage of apoptotic and necrotic cells is shown (G-J). Data represents mean ± SD. (*) $p < 0.05$; (**) $p < 0.001$; (***) $p < 0.0001$ when compared to negative control by one-way ANOVA and Dunnett's post-test. SSC = Side Scatter (granularity); FSC = Forward Scatter (relative size); FITC and PE = Channels; AV = Annexin V; PI = Propidium Iodide; LP = Low population; HP = High population; IC_{50} = 50% maximal Inhibitory Concentration.

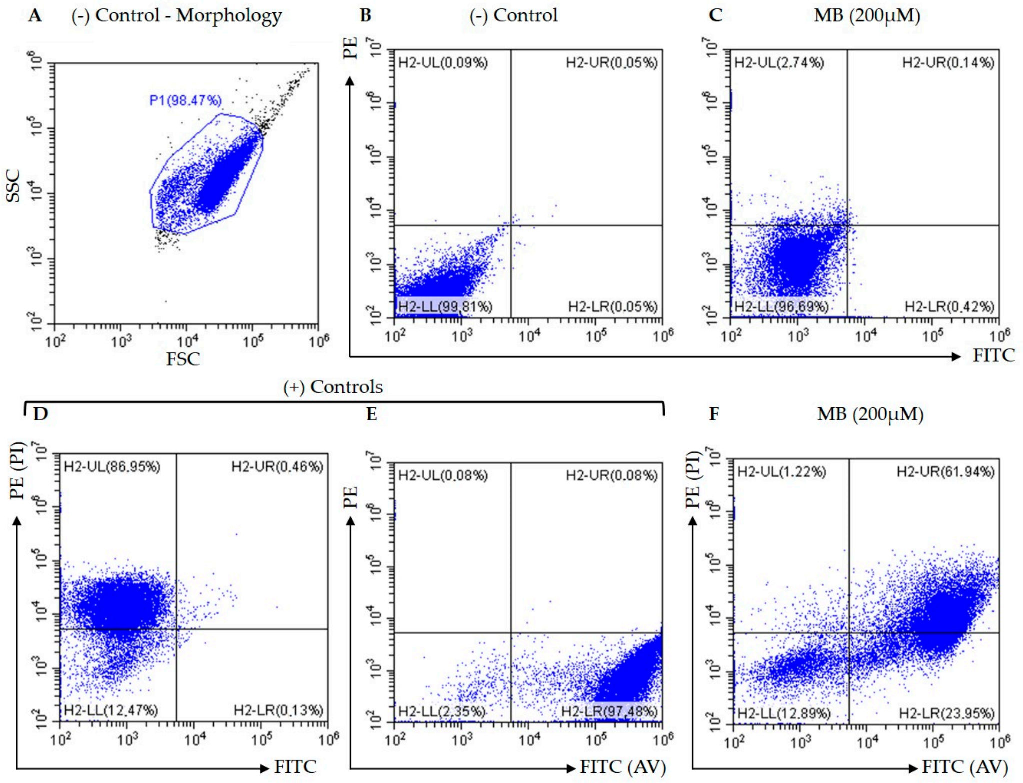

Figure S5. Cell death evaluation by flow cytometry on promastigote forms of *Leishmania amazonensis* PH8 strain (10^6 parasites/mL) exposed to 200 μM of methylene blue B (MB) for 24 h and labeled with AV and PI (F). Untreated parasites were used as negative control (A-B). Untreated and killed parasites by heating at 60 °C for 15 min were used as positive control (D-E). SSC = Side Scatter (granularity); FSC = Forward Scatter (relative size); FITC and PE = Channels; AV = Annexin V; PI = Propidium Iodide.

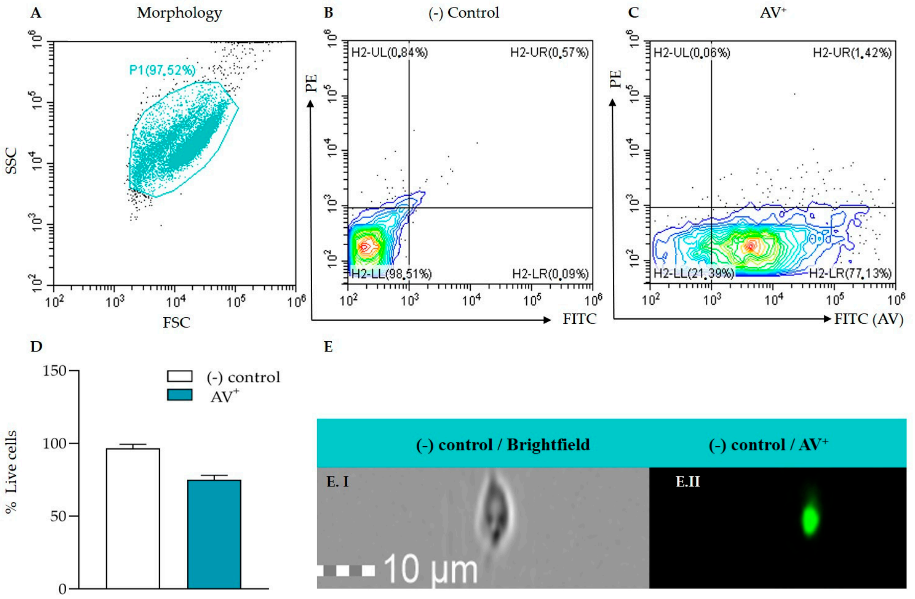

Figure S6. Phosphatidylserine exposure in *ex vivo* amastigote forms of *Leishmania amazonensis* LTB0016 strain (10^6 parasites/mL) labeled with AV (C-D). Unlabeled parasites were used as negative control (B). The percentage of live cells and images flow cytometry (Ammnis Stream MK XII) are shown (D-E). Data represents mean ± SD. SSC = Side Scatter (granularity); FSC = Forward Scatter (relative size); FITC and PE = Channels; AV = Annexin V.

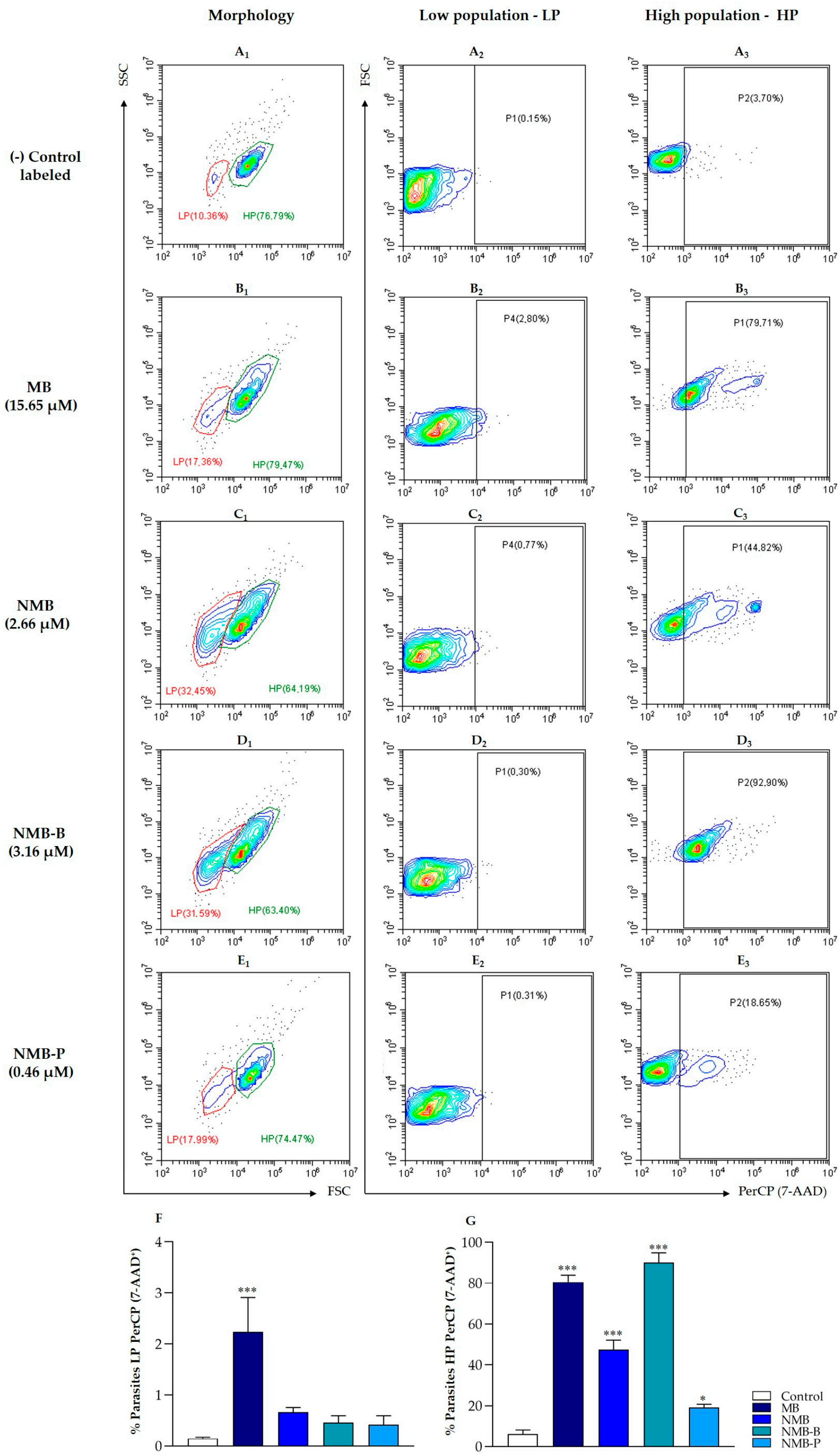

Figure S7. Cell death evaluation by flow cytometry on subpopulations of *Leishmania amazonensis* ex vivo amastigote forms (10^6 parasites/mL, LTB0016 strain) exposed to IC_{50} of methylene blue (MB – Panel B), new methylene blue (NMB – Panel C), new methylene blue B (NMB-B – Panel D), and new methylene blue P (NMB-P – Panel E) for 24 h. Samples were labeled with 7-AAD. Untreated parasites 7-AAD$^+$ were used as negative control (Panel A). The percentage of necrotic cells is shown (F-G). Data represents mean ± SD. (*) $p < 0.05$; (**) $p < 0.001$; (***) $p < 0.0001$ when compared to negative control by one-way ANOVA and Dunnett's post-test. SSC = Side Scatter (granularity); FSC = Forward Scatter (relative size); PerCP = Channel; 7-AAD = 7-Amino-Actinomicina D; LP = Low population; HP = High population; IC_{50} = 50% maximal Inhibitory Concentration.
